# Mesenchymal stem cells for critical limb ischemia: their function, mechanism, and therapeutic potential

**DOI:** 10.1186/s13287-022-03043-3

**Published:** 2022-07-26

**Authors:** Laura V. Lozano Navarro, Xueyi Chen, Lady Tatiana Giratá Viviescas, Andrea K. Ardila-Roa, Maria L. Luna-Gonzalez, Claudia L. Sossa, Martha L. Arango-Rodríguez

**Affiliations:** 1 Faculty of Health Sciences, Universidad Autónoma de Bucaramanga (UNAB), 681004153 Bucaramanga, Colombia; 2 Banco Multitejidos y Centro de Terapias Avanzadas, Fundación Oftalmológica de Santander–FOSCAL, 681004153 Floridablanca, Colombia; 3 Programa Para el Tratamiento y Estudio de Enfermedades Hematológicas y Oncológicas de Santander (PROTEHOS), 681004153 Floridablanca, Colombia; 4 Universidad de Valencia, Valencia, Spain

**Keywords:** Critical limb ischemia, Revascularization, Limb amputation, Mesenchymal stem cells, Blood vessels

## Abstract

Peripheral arterial disease is atherosclerotic occlusive disease of the lower extremity arteries and afflicts hundreds of millions of individuals worldwide. Its most severe manifestation is chronic limb-threatening ischemia (Petersen et al. (Science 300(5622):1140–2, 2003)), which is associated with severe pain at rest in the limbs, which progresses to necrosis, limb amputation, and/or death of the patient. Consequently, the care of these patients is considered a financial burden for both patients and health systems. Multidisciplinary endeavors are required to address this refractory disease and to find definitive solutions that lead to improved living conditions. Revascularization is the cornerstone of therapy for preventing limb amputation, and both open vascular surgery and endovascular therapy play a key role in the treatment of patients with CLI. Around one-third of these patients are not candidates for conventional surgical treatment, however, leading to higher amputation rates (approaching 20–25% at one year) with high morbidity and lower quality of life. Advances in regenerative medicine have enabled the development of cell-based therapies that promote the formation of new blood vessels. Particularly, mesenchymal stem cells (MSCs) have emerged as an attractive therapeutic agent in various diseases, including CLI, due to their role in tissue regeneration and immunomodulation. This review discusses the characteristics of MSCs, as well as their regenerative properties and their action mechanisms on CLI.

## Introduction

Critical limb ischemia [1] is the most advanced stage of peripheral arterial disease (PAD) [2]. It has been reported that 10% of patients with PAD may have CLI, and 5–10% of patients with asymptomatic PAD or intermittent claudication will progress to CLI over five years [3]. The estimated total number of patients with CLI in the USA, Europe, and Japan is approximately 6.5 million [4]. CLI prevalence in the US population above 40 years old is estimated to be 1.28%, which is approximately 2 million total CLI patients in this country, with an annual incidence range from 0.26 to 0.48%. Amputation rates may vary among patients in terms of severity of illness, comorbidities, and other sociodemographic conditions but are consistently high in most studies, typically exceeding 15–20% in the first year and reaching values of up to 67.3% at four-year follow-up in patients with more advanced disease [5]. This ultimately affects not only limb loss but also in-hospital and long-term mortality, which over five years is usually above 50% [6].

These patients also suffer a significant reduction in quality of life due to permanent local wound treatment and the chronic use of pain-relieving medications, plus other comorbidities, leading to a dependency on the support of caregivers. The poor clinical outcomes in these patients result in the increased use of medical resources, and high hospitalization rates of up to 375,000 admissions annually, leading to a considerable economic burden for national health care systems [6, 7]. In this context, Mustapha et al. analyzed data from US Medicare beneficiaries for four years after diagnosis and estimated a cost per CLI patient of between 93,800 USD and 117,800 USD, although this does not represent an overall national estimate, which could be several times higher [8].

Currently, standard therapeutic options include revascularization using a surgical or endovascular approach, depending on the patient's comorbidities, their vascular anatomy, and the location of the vascular lesions [9]. Multidisciplinary endeavors are required to address this refractory disease, in order to find definitive solutions that will lead to improved living conditions. New strategies for regenerative medicine have enabled the development of therapeutic angiogenesis through stem cells, recombinant proteins, and gene transfer [10, 11].

Stem cells have thus emerged as an attractive therapeutic agent in various diseases, including CLI, due to their angiogenic role, and their regenerative and immunomodulatory effects on tissue lesion. Autologous bone marrow stem cells (a-BM-SC) are considered the gold standard of cell therapy for CLI, but this therapy has several disadvantages that limit its use, such as the cardiovascular risk pattern common to CLI patients, and complications arising from invasive aspiration procedures. The angiogenic potential of transplanted cells also directly depends on the characteristics of the donor, which in this particular case may be impaired by the age and general health of CLI patients, and so a-BM-SC may not be the best therapeutic option for this condition [12]. Other stem cell sources have been explored to overcome these obstacles. Mesenchymal stem cells (MSCs) are a particularly attractive therapeutic agent for treating CLI. MSCs have outstanding advantages over the other stem cell populations, they can be obtained from healthy allogeneic donors, present low immunogenicity (reduced expression of MHC class II constitutive molecules), have anti-inflammatory properties, and are relatively simple to grow and expand in vitro [13, 14]. These characteristics have recently encouraged the development of preclinical and clinical trials for the treatment of ischemic disorders, including stroke, coronary artery disease, and CLI [15]. The goal of this review is to highlight the features, functions, and mechanisms of action of MSCs in the context of therapeutic angiogenesis for CLI.

## Characteristics of MSCs

### MSC tissue sources, isolation, and expansion

MSCs are a heterogeneous subset of stromal cells distributed throughout the stroma of almost all tissues/organs in vivo [16], giving rise to a variety of sources for their isolation, including adult tissue (e.g., bone marrow (BM), peripheral blood, and adipose tissue (AD)), as well as fetal (e.g., umbilical cord blood (UCB), Wharton’s jelly (WJ), amnion, amniotic fluid, and placenta) and embryonic tissues [16, 17]. Their cellular concentrations in tissue are low, therefore, requiring a large in vitro expansion for their subsequent therapeutic use [18]. Despite the many sources, most of the MSCs used for clinical trials are primarily derived from BM, AD, UCB, and WJ of which BM is considered the gold standard [17]. Nevertheless, BM-MSC isolation involves a highly invasive aspiration procedure that often causes severe pain and has a high risk of infection [19]. Particularly, a limited volume of BM is also collected at any one time, resulting in a low MSC yield, which appears to be detrimental to the potential for MSC proliferation and differentiation, as indicated by the presence of senescence [20]. Other novels MSC sources have therefore been explored [19], including cadaveric MSCs from BM [21] and menstrual blood-derived stem cells [22].

MSC isolation methods vary depending on their source: BM-MSCs are usually isolated using the density gradient procedure, or by direct cell plating on a solid surface due to their adhesion capacity [23], while AD-MSCs and WJ-MSCs are obtained by collagenase digestion and density gradient separation [25, 26].

On the other hand, fetal bovine serum (FBS) is the supplement to cell culture media more commonly used [24]. Nevertheless, serum-free media formulations have been developed in the last decades, particularly in good manufacturing practice guidelines that need to be followed to use these cells in cell-based therapy treatments. In order to decrease their use, many alternatives have been developed as human components such as human serum, platelet-rich plasma, and human platelet lysate [24], and numerous studies have reported its potential effect in promoting MSC proliferation, relative to FBS [25–28].

### Minimal criteria for MSC characterization

**Fig. 1:**
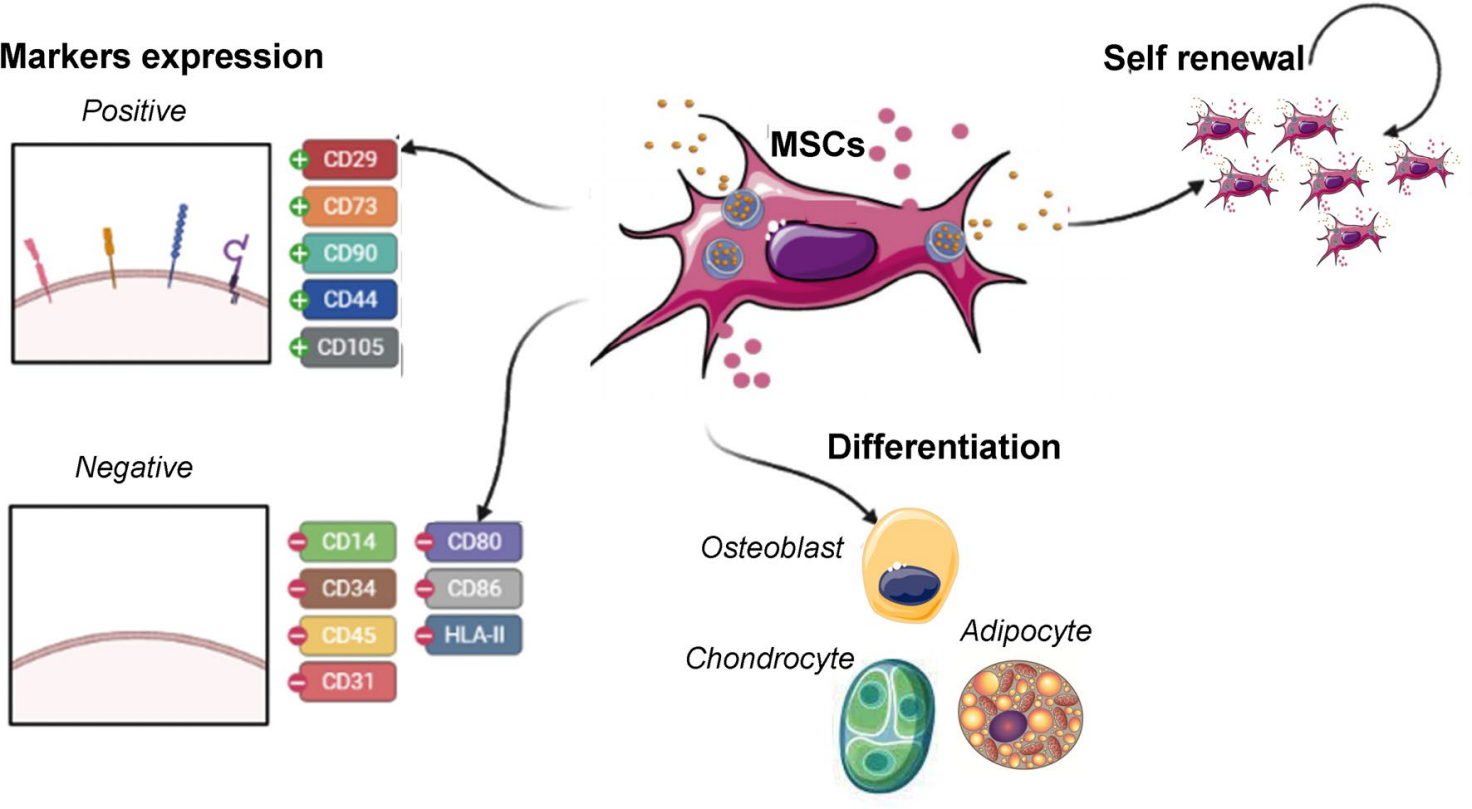
Minimal criteria for MSC characterization. MSCs are plastic-adherent and spindle-shaped morphology, they must be capable to differentiate into adipocytes, chondroblasts, and osteoblasts in vitro, and MSCs must be positive for surface antigen markers (CD29, CD73, CD90, CD44, and CD105) and they must lack expression of antigen markers (CD31, CD14, CD34, CD45), and costimulatory molecules (CD80, CD86), and HLA-DR surface molecules

The International Society for Cell Therapy (ISCT) released a set of minimal criteria for laboratory-based scientific investigations [29]. These guidelines include (i) MSCs are plastic-adherent and display a spindle-shaped morphology during standard culture conditions, (ii) MSCs must be capable to differentiate into adipocytes, chondroblasts, and osteoblasts in vitro*,* and (iii) MSC population must be positive (≥ 95%) for surface antigen markers such as CD29, CD73, CD90, CD44, and CD105, and MSCs must lack expression (≤ 2% positive) of endothelial markers (CD31), hematopoietic markers (CD14, CD34, CD45), human leukocyte antigen (HLA) class II, costimulatory molecules (CD80, CD86), and HLA-DR surface molecules [30], although these markers may also vary among different MSC sources (e.g., UCB-MSCs *vs*. BM-MSCs) [31, 32, 34, 35] (Fig. 1).

### MSC delivery, homing, and engraftment capacity on CLI

#### MSC delivery

Although it has been demonstrated that MSCs play a role in the angiogenic process on CLI, there is not currently a recommended approach for delivering MSCs as a treatment for this condition. Local administration is the most common route through which MSCs are applied, particularly intramuscular (into the gastrocnemius muscle) or intravascular (along the occluded native arteries in parallel orientation to the axial arteries) [33]. Systemic administration (intravenous (IV) or intra-arterial (IA)) is less commonly used [34].

There is still no clear consensus regarding the differential therapeutic effects of each route of administration [35]. Indeed, some studies have shown that one advantage of intramuscular administration is the ability to deliver MSCs directly to the site of the lesion, and the creation of local depots of MSCs with increased local paracrine activity and local release of arteriogenic cytokines, such as vascular endothelial growth factor (VEGF), basic fibroblast growth factor (bFGF), placental growth factor (PlGF), and monocyte chemoattractant protein-1 (MCP-1) [36]. Similarly, Dong et al. [37] showed a significantly improved ankle–brachial pressure index (ABPI) and transcutaneous partial pressure of oxygen (TcPO_2_) after intramuscular injections of MSCs, results that were not obtained when cell therapy was performed through intra-arterial injections; however, no significant differences were reported between the routes of administration as regards significant pain relief and pain-free walking distance. It is also reported that although direct injection increases the localization of MSCs in their target tissue, it does not improve engraftment or the survival rate; this route can also cause further tissue damage from the bolus injection.

Systemic administration (either IV or IA delivery) is a minimally invasive procedure that allows the wide distribution of cells throughout the body [34]. However, MSCs must migrate from the blood circulation to the target tissue to achieve their therapeutic effect. MSCs have been reported to express molecules such as very late antigen-4 (VLA-4) and vascular cell adhesion molecule-1 (VCAM-1), which modulate vascular endothelial cell adhesion and transendothelial migration. In addition, through stimulation of certain cytokines and proteolytic enzymes, such as matrix metalloproteinases (MMP-2 and MMP-9), degradation of the basement membrane is carried out for tissue invasion. Overall, this mechanism implies a complex process that is also coordinated by cytokine stimulation [38].

**Fig. 2:**
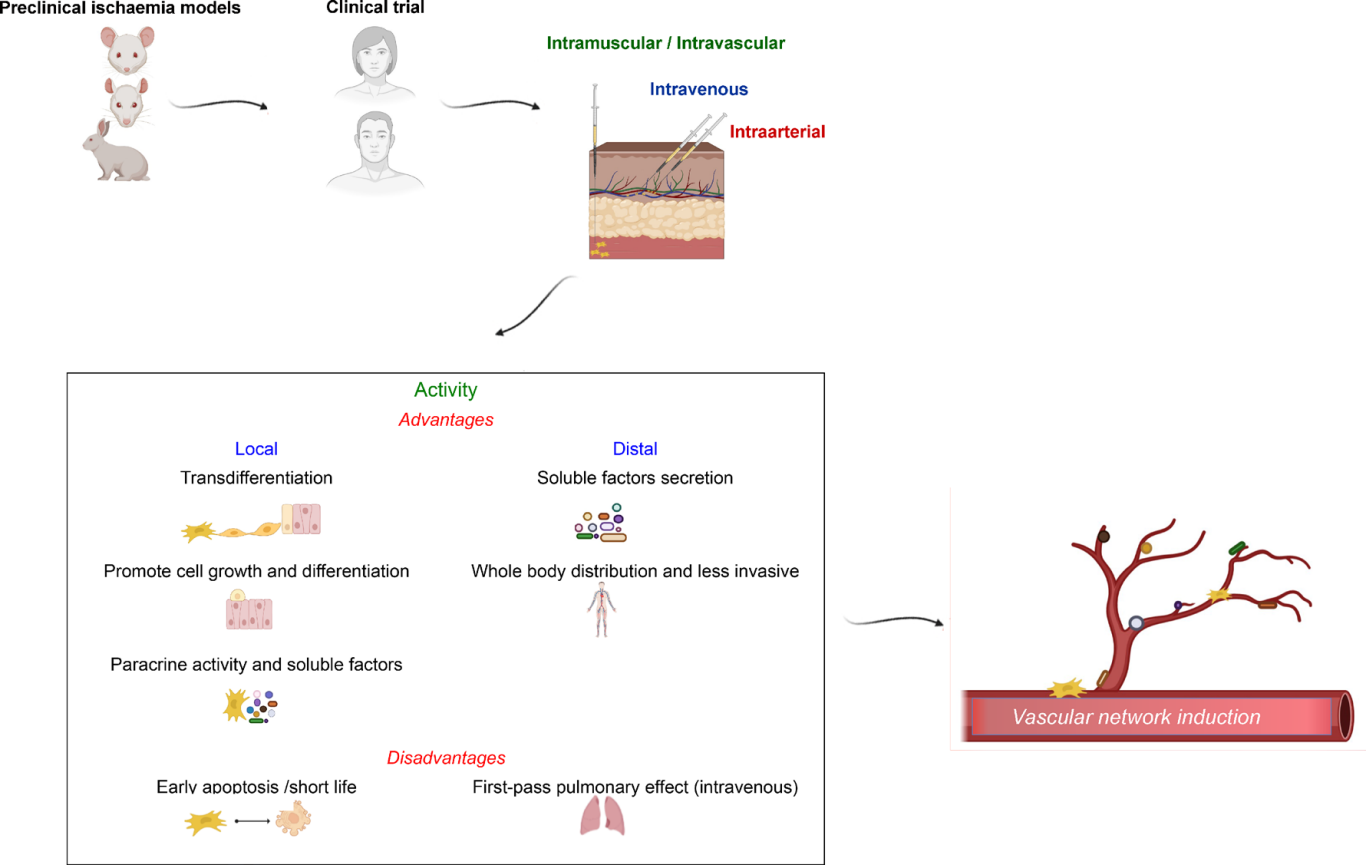
MSC routes of administration in CLI: advantages and disadvantages

Although IV delivery is the easiest and the most common systemic route in clinical practice, a frequently associated problem is the so-called pulmonary “first-pass” effect, which results in the significant entrapment of cells, leading to a higher absolute number of cells needed to ensure that a minimum number of cells reach the injury site distal to the lungs [34]. The cause of this entrapment in the lungs is probably a combination of mechanical and physiological conditions and may be due to the small capillary size, large capillary network, and strong adhesion properties of MSCs. On the other hand, IA administration avoids the lung's route at least once, reducing the “first-pass pulmonary effect” and allowing a reduction in the cell dose [34] (Fig. 2).

The promotion of vascularization and angiogenesis is fundamental for efficient organ reconstitution and replacement [39]; therefore, another modality for transplantation of MSCs includes scaffolds and growth-stimulating signals that provide the structural support for cell attachment and subsequent tissue development. Tissue engineering builds an adequate environment for the delivery, aligning and maintaining cell connections in favor of vascularization and angiogenesis upon implantation. Based on the tissue compatibility, scaffolds can be natural or synthetic, being the synthetic biomaterials easier to control. Some of the different biomaterials that have been used and developed for tissue-engineered approaches are collagen, elastin, Matrigel, fibrin, alginate, chitosan, and agarose [40].

Other describe strategies that intensify angiogenesis potential include genetic manipulation and conjugation of pro-angiogenic factors [41]. miRNA therapy has been also described as a scaffold-base therapy, playing an important role in the induction/inhibition of angiogenesis [42, 43].

Despite the remarkable intrinsic properties of MSCs for the treatment of CLI, there is still a lack of standardized routes and delivery methods to guarantee MSC optimal engraftment. Controlled studies may therefore be required to investigate appropriate approaches to delivering MSCs and ensure their survival at the ischemic sites.

#### MSC homing and engraftment

Some preclinical studies on the hindlimb ischemia model have shown MSC homing and engraftment by using local (intramuscular) or systemic routes. In particular, Lee et al. [44] labeled human adipose-derived MSCs (hAD-MSCs) with dye-tagged dibenzyl cyclooctyne (DBCO-Cy5-hAD-MSCs) to track the grafted cells and investigate their direct action and migration pattern at the inner thigh in the ischemic hindlimb mice model. After intramuscular administration of the 5 × 10^4^ DBCO-Cy5-labeled, cells were monitored for two weeks using a 360º fluorescence tomographic imaging system. The authors found that the DBCO-Cy5-hAD-MSCs appeared to gradually converge at the inner thigh in the ischemic hindlimb, indicating cell migration toward the ischemic lesions; in contrast, a certain amount of the signal was initially observed but quickly disappeared in the normal hindlimb. These findings were confirmed by histological analysis two weeks post-transplantation, where DBCO-Cy5-hAD-MSCs were found in ischemic tissue, indicating the integration of the labeled cells into the host tissue [44].

Similarly, Iwase et al. [45] used an animal model of hindlimb ischemia with male Lewis rats who received rat bone marrow-derived MSCs (rBM-MSCs) (5 × 10^6^ cells) or rat bone marrow-derived mononuclear cells (rBM-MNC) (5 × 10^6^ cells) to demonstrate the presence and viability of rBM-MSCs in the interstitial tissues three weeks after intramuscular injection, and the majority of rBM-MNC revealed severe organelle damage and disintegration. rBM-MSCs and rBM-MNC were also labeled with a fluorescent dye (PKH26 red fluorescent cell linker) and then transplanted into the ischemic thigh muscle in rats to examine cell differentiation. This subgroup of rats was euthanized three weeks after rBM-MSCs or rBM-MNC transplantation, and tissue sections were incubated with anti-von Willebrand factor (vWF) or anti-alpha-smooth muscle actin (α-SMA) antibodies, and endothelial and vascular smooth muscle cells markers, respectively. Histological studies revealed that PKH26-positive cells expressed vWF in both the rBM-MSCs and rBM-MNC groups, although quantitative analysis demonstrated that the number of PKH26/vWF-double-positive cells was significantly higher in the rBM-MSCs group than in the rBM-MNC group. In contrast, some of the transplanted rBM-MSCs were positive for α-SMA, but none of the rBM-MNC was stained for this antibody. rBM-MSCs thus survived well under an ischemic environment and differentiated not only into endothelial cells but also vascular smooth muscle cells.

Xie et al. [46] evaluated the potential effects of human placenta-derived mesenchymal stem cells (hPMSCs) on mouse hindlimb ischemia. hPMSCs were labeled with a fluorescent dye (CM-DiI-hPMSCs) and delivered via intramuscular injection (5 × 10^5^ cells) into male C57BL/6 J mice. The mice had previously been intravenously injected with green fluorescence identified FITC-UEA-l to enhance the contrast of functional perfused vessels, and to test whether the vascular networks had connected to the mouse circulation. Ischemic hindlimbs treated with labeled hPMSCs were isolated and analyzed by fluorescent microscopy at Day 14. The merged images of both stainings (FITC-UEA-l and CM-DiI-labelled hPMSCs) showed the incorporation of hPMSCs into murine vascular networks or capillary networks, indicating their participation in angiogenesis in vivo. Immunostaining also showed that anti-human CD31 and anti-human α-SMA cells were detected in hPMSC-treated tissues after 21 days, indicating the endothelial and smooth muscle cell differentiation of hPMSCs in the ischemic limbs.

Huang et al. had similar results when comparing rBM-MSCs obtained from male C57BL/6 J (B6) and Balb/c mice cultured under hypoxic *vs.* normoxic conditions; the cells were labeled with carboxyfluorescein diacetate succinimidyl ester (CFSE), and the findings demonstrated the presence of CFSE-labelled cells in the ischemic tissue of mice receiving hypoxic rBM-MSCs, but not in the tissue of the mice that received normoxic rBM-MSCs at seven days post-transplantation, implying that hypoxia can further ameliorate blood flow by enhancing engraftment. A long-term tracking assay (four weeks post-transplantation) using double immunofluorescence for bromodeoxyuridine (BrdU) and CD31^+^ (endothelial cell marker) revealed that some of these BrdU^+^ cells were observed in the CD31^+^ blood vessels, indicating that some transplanted cells were incorporated into neo-vessels, and indeed functioned and contributed to blood perfusion. Some were also positive for α-SMA or desmin in the ischemic regions, also implying that some transplanted cells differentiated into muscle tissues [47].

It has also been reported that the homing process and engraftment depend on the MSC immunomodulatory capacity, which can be reduced by certain pathologic conditions such as diabetes, since hyperglycemia-mediated down-modulation of chemokine receptor expression in endothelial progenitor cells and other progenitor cells, resulting in defective angiogenesis and impaired reparative responses [48].

Some preclinical studies have evidenced a shorter settlement time at the ischemic site after local administration [34, 49]; however, the number of cells tends to decrease progressively [50, 51]. It has been reported that many transplanted cells can undergo apoptosis at an early stage [52], suggesting a survival period long enough to induce angiogenesis in other ways [53]. Cumulatively, these results suggest that transplanted MSCs survive after local or systemic administration, engraft into the ischemic tissue, and subsequently induce vascular networks.

## Molecular mechanisms associated with the clinical potential of MSCs

MSC angiogenic properties have been studied for a long time, but some of the underlying mechanisms of action remain unclear. MSCs belong to a special population of cells with homing ability, meaning they can selectively migrate to ischemic sites regardless of the delivery method in response to a variety of signals secreted by injured and immunological cells. Evidence suggests that MSCs can potentially move from their niche into the peripheral circulation and pass through vessel walls to reach target tissues. Once in the target site, they exert their effects either directly or through the secretion of paracrine factors [54].

### Cell differentiation and/or transdifferentiation

Usually, MSCs retain the ability to differentiate into a variety of mesenchymal lineages, including bone, cartilage, tendon, fat, bone marrow stroma, and muscle, induced by specific medium conditions such as growth factors and cytokines [55]. After delivery, the cell differentiation mechanism includes MSC migration to ischemic sites in response to chemotactic signals in vivo [56]. Once MSCs are located at these sites, they start to engraft, differentiate and/or transdifferentiate to actively participate in tissue regeneration [57]. In the same way, numerous evidence has shown that part of their angiogenic potential comes from their ability to differentiate directly into blood vessel components, such as endothelial cells (EC), which under hypoxic conditions secrete multiple angiogenic factors, such as VEGF, which plays an important role in cell survival, proliferation, and migration [46].

Although some studies have demonstrated the differentiation and/or transdifferentiation of MSCs in ischemic tissue [58–60], there is evidence of poor engraftment, particularly in allogeneic transplantation, which could be due to an immune rejection despite MSC immunomodulatory properties [50]. Indeed, Zangi et al. carried out preclinical experiments in mice, comparing the in vitro immunomodulatory capacity of mBM-MSCs *vs.* fibroblasts, and observing that mBM-MSCs prevented the proliferation of CD4^+^ and CD8^+^ T cells, while fibroblasts did not produce significant suppression in either of the two immunological lineages. They subsequently evaluated the in vivo survival of luciferase-labeled mBM-MSCs (Luc^+^mBM-MSCs) in immunocompetent allogeneic recipients *vs.* immune-deficient recipients (Balb-Nude or non-obese diabetic/severe combined immunodeficiency (NOD-SCID)). The analysis showed that Luc^+^mBM-MSC survival was significantly shorter in immunocompetent allogeneic recipients compared to that exhibited in immune-deficient recipients. These results demonstrate that under allogeneic conditions, mBM-MSCs cannot completely evade the immune system or induce immune memory and potential rejection [52].

On the other hand, Guo et al. explored whether EC differentiation from human AD-MSCs (EC-hAD-MSCs) was effective in improving therapeutic outcomes in the treatment of ischemic disease. In this study, hAD-MSCs were cultured under EC differentiation medium for 10 days. Flow cytometry analysis, western blot, and reverse transcription-polymerase chain reaction (RT-PCR) confirmed the EC-specific markers EC-hAD-MSCs relative to undifferentiated adipose MSCs (UA-hAD-MSCs). In vitro angiogenic studies showed the ability of UA-hAD-MSCs to express significantly higher levels of representative pro-angiogenic genes, chemokines, and growth factors than EC-hAD-MSCs. Analyses of engrafted cells in hindlimb sections after UA-hAD-MSC or EC-hAD-MSC injection marked with red fluorescent protein were carried out using NOD/severe combined immunodeficiency mice. Laser Doppler perfusion image (LDPI) analysis was performed, revealing a greater recovery of blood perfusion in the limbs injected with UA-hAD-MSC compared to those injected with EC-hAD-MSC. Vascular and capillary density in the ischemic hindlimb adductor muscle after cell injection was also measured using two endothelial markers (isolectin B4 (ILB4^+^) and CD31^+^). The outcomes revealed that the UA-hAD-MSC group induced significantly higher capillary density than EC-hAD-MSCs or a control group. Four weeks after transplantation, tissue was harvested and immunohistochemistry analysis revealed that the UA-hAD-MSCs group showed significantly higher levels of the representative pro-angiogenic genes, chemokines, and growth factors than the EC-hAD-MSCs group, as well as higher adhesion capacity, increased engraftment potential, and higher recovery of blood perfusion according to LDPI [61]. These results support the idea that the differentiation of hAD-MSCs does not improve their angiogenic potential and thus may not be the primary mechanism by which angiogenesis occurs.

### Paracrine signals

**Fig. 3:**
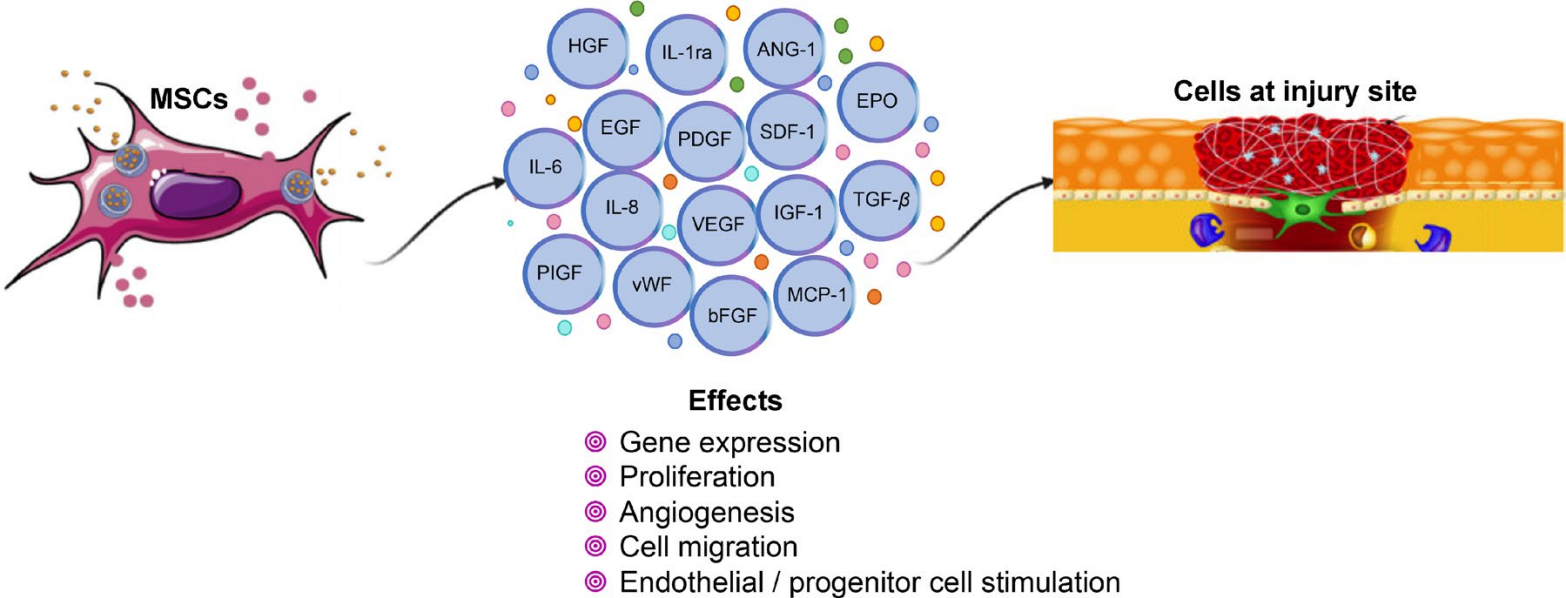
MSC paracrine activity performs its angiogenic modulation through a complex synergic activity between many bioactive molecules generating profound effects on cellular dynamics and improving the regenerative response

Paracrine activity has been reported as the principal mechanism for the MSC therapeutic effects, mainly through the secretion of growth factors that actively contributes to promoting vascularization processes, leading to an improvement in tissue repair [62–66]. The secretome, known as the set of elements released from cells including cytokines, growth factors, enzymes, microparticles, miRNAs, and extracellular vesicles (exosomes), allows the transference of proteins, lipids, and genetic material to recipient cells, generating profound effects on cellular dynamics and improving the regenerative response [67, 68]. Several studies have identified the therapeutic effects mediated by exosomes as impairment for neoplastic transformation, ability to induce angiogenesis, regeneration, the proliferation of epithelial [69], immunomodulatory effect by downregulation of interferon-γ secretion [70], and wound healing via cell proliferation and keratinocyte migration [71]. Additionally, MSC-derived exosomes have shown high stability in the body, ability for modification with targeted molecules, high protein loading capacity [72], and different miRNA expression patterns depending on the age of the donor [73] (Fig. 3).

The MSC factors that contribute to angiogenesis, tissue regeneration, and endothelial/progenitor cells stimulation on CLI are insulin growth factor-1 (IGF-1), VEGF, bFGF, transforming growth factor-beta (TGF-*β*), vWF, angiogenic factors CD31, stromal-derived factor-1 (SDF-1), angiopoietin-1 (ANG-1), erythropoietin, platelet-derived growth factor (PDGF), placental growth factor, interleukin-8 (IL-8), IL-6, hepatocyte growth factor (HGF), epidermal growth factor (EGF), MCP-1, macrophage colony-stimulating factor (M-CSF), interleukin-1 receptor antagonist (IL-1ra), and macrophage inflammatory protein-1alpha and beta, among others [74, 75].

Indeed, several studies have shown that the conditioned medium (CM) derived from MSCs has a great impact on the activation of different endothelial cell responses at injury sites, promoting angiogenesis and functional recovery [76].

One mechanism that favors the increased paracrine effects that promote angiogenesis is the activation of the AKT signaling pathway. Chang et al. showed the AKT phosphorylation in an endothelial cell line (HAECs) by E69E7-MSCs conditioned medium (E6E7-CM) increasing the expression and release of IL-1β and VEGF-A in vitro. An ischemic model in Balb/c mice subsequently showed that E6E7-CM ameliorates limb loss and improves muscle fibrosis and endothelial density in ischemic limbs [76].

A study by Lee involving hAD-MSCs treated with TNF-α showed that they secrete several proteins, growth factors, cytokines, proteases, and protease inhibitors in TNF-α-CM. The intramuscular injection of TNF-α-CM in the simulated chemotactic migration and in vivo homing of human endothelial progenitor cells (EPCs) promoted angiogenesis in the ischemia limb through IL-6 and IL-8 dependent mechanisms, which improved blood perfusion and inhibited tissue necrosis in the ischemia hindlimbs [77]. These events led to a decrease in the number of proliferating cells, and an increase in the number of vWF-positive capillaries and α-SMA-positive arteries/arterioles in the ischemic limbs. When TNF-α-CM was applied topically, acceleration in the re-epithelialization, proliferation, and angiogenesis was observed. These results suggest that TNF-α-CM can be used for neovascularization and regeneration in peripheral artery disease [77].

Recent studies have shown that bone marrow-derived EPCs contribute to ischemic tissue repair by secreting paracrine factors. Liew et al. identified different angiogenesis-related factors in the CM of MSCs derived from B6 and C57BKS mice, such as matrix metalloproteinase (MMP)-3, C-X-C motif chemokine ligand (CXCL)-16, CXCL-4, CINC-10, insulin-like growth factor binding protein (IGFBP)-3, monocyte chemoattractant protein (MCP)-1, serpin e1, MMP-9, IGFBP-2, IGFBP-9, tissue inhibitor of metalloproteinases (TIMP)-1, pentraxin-3, and VEGF [75]. These factors have been related to the modulation of several essential cellular processes, such as cell migration, senescence, autophagy, proliferation, survival, and angiogenesis.

On the other hand, several studies have discussed the role of cell-to-cell interactions between MSCs and EC in angiogenesis and tissue regeneration. In animal models has been observed that once MSCs delivered, they are recruited toward ischemic tissue by chemostatic signaling and express a variety of specific cell surface molecules such as integrins, which regulate the rolling and adhesion of MSCs to EC. Later MSC transmigration into the vessel wall is mediated by platelet-endothelial cell adhesion molecule-1 (PECAM-1/CD31), junctional adhesion molecules such as VCAM-1, and cadherins, similar to leukocyte mechanisms. It has been described that soluble factors or lipid vesicles secreted by MSCs into the microenvironment play an important role in cross-talk, transfer of information, EC survival, transdifferentiation into EC, and mobilization of EPCs from the bone marrow [78]. On the other hand, Chen et al. reported that MSCs have the potential to stabilize vascular endothelium injuries (paracellular and transcellular permeability) by paracrine mechanisms, particularly related to HGF secretion and its effect on the expression of binding proteins, remodeling of endothelial junctions, and EC proliferation [79].

#### Immunomodulation effect

The immunomodulatory effect of MSCs has been reported in many studies and is mediated by paracrine mechanisms [80]. MSCs also exert immunomodulatory effects by inducing neighboring cells to secrete anti-inflammatory cytokines [9], which may be useful in inhibiting excessive inflammation. MSC administration has also been shown to reduce the levels of TNF-α alpha in vitro, a major pro-inflammatory cytokine. Numerous data on a wide range of pathological conditions demonstrate that MSCs exert potent cytoprotective and anti-apoptotic actions through the release of soluble active mediators in a hypoxic MSC-conditioned medium, which can reduce apoptosis and necrosis when exposed to low oxygen tension [3].

The nature of the signals involved in the immunomodulatory effect of MSCs has been studied in several in vitro and animal models. Hypoxia-inducible factor-1 (HIF-1) is a key mediator of the hypoxic response complex. It regulates the transcription of several types of genes under hypoxic conditions related to chemokine secretion; the most important of these signals are SDF-1 and HGF, which are up-regulated during tissue damage [81]. With specific regard to the SDF-1 axis, CXCR4/7 functions as a cognate receptor expressed on the MSC surface and is considered a key link in the homing process of stem cells. Under normoxic conditions, proline hydroxylation induces conformational changes in the HIF-1α subunit due to its binding to the von Hippel–Lindau (VHL) protein and can subsequently be rapidly degraded by ubiquitin/proteasome pathways. LincRNA-p21, however, a large intergenic non-coding RNA located on chromosome 21, is induced by HIF-1α under hypoxic conditions, which disrupts the HIF-1α-VHL interaction, inhibiting HIF-1α degradation and leading to its stability in target tissues. MSCs induced by hypoxic preconditioning resulted in the increased expression of LincRNA-p21, HIF-1α, and CXCR4/7, supporting their migration-related function and homing capacity [82].

The immunomodulatory effect of MSCs is communicated via MSC-secreted cytokines and has been proven to rely on the local microenvironment, as some effects depend on the pre-treatment of MSCs with inflammatory cytokines. These cytokine-mediated effects suggest a key role for regulatory T cells and monocytes in the overall pattern [83]. MSCs can affect several cells, such as macrophages, NK cells, B cells, T cells, immature dendritic cells, and mature dendritic cells. These angiogenic mechanisms participate in the reduction of cell death, improving the regeneration and function of tissues [84].

The infiltration of neutrophils, macrophages, dendritic cells, and T cells not only contributes to chronic inflammation but also causes the release of elastase enzyme, which causes the inhibition of important healing factors such as PDGF and TGF-β [85]. Liu et al. found that macrophage migration ability was improved by ASCs under hypoxia conditions. Their results showed that ischemic muscle increased macrophage infiltration after ASC injection [86]. ASCs may have an immunoregulatory effect on ischemic muscle through the enhancement of macrophage migration and induction of macrophages recruited to the M2 phenotype, showing that M2 macrophages were induced by ASCs through activation of the IL-10/STAT3 pathway, as per other reports of M2c polarization [87]. M2 macrophages in ASC-treated mice thus resemble the M2c subtype, indicating the vital role of M2c macrophages in ASC-mediated ischemic muscle repair [86].

**Fig. 4:**
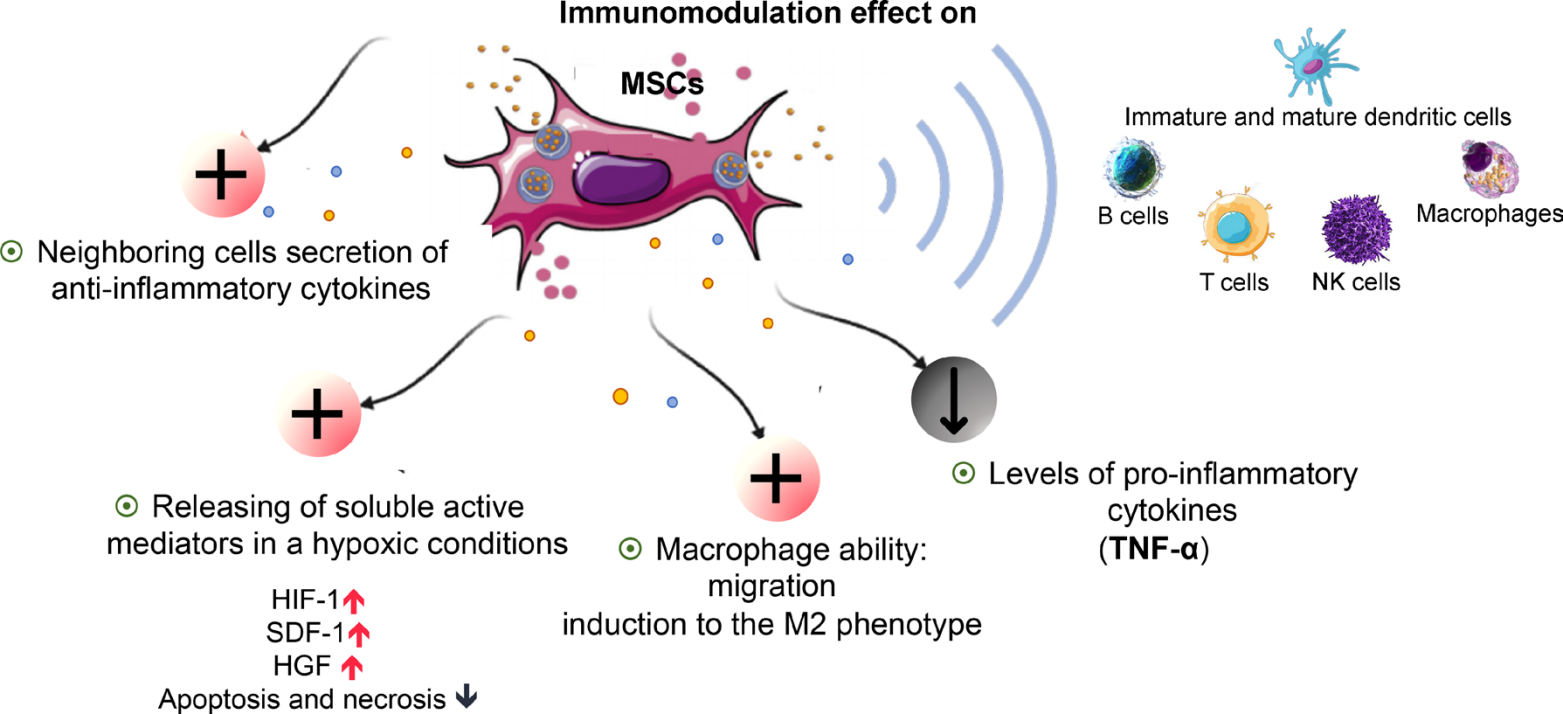
MSC immunomodulatory effect operates through a synergy of cell contact-dependent mechanisms and soluble factors

It remains unclear whether the phagocytosis of living MSCs occurs via the innate immune cells of the host, or whether the MSCs must undergo apoptosis to subsequently perform phagocytosis. Galleu et al. have shown that infused living MSCs are subject to perforin-induced apoptosis through recipient cytotoxic cells [88]. Heat-inactivated MSC or fragmented-MSC thus most likely does not carry out changes in their immunomodulatory characteristics under different environmental stimuli (Fig. 4).

#### Transfer of mitochondria

Recent studies have shown that intercellular communication using tunneling nanotubes can transfer mitochondria between neighboring cells. For example, MSCs have recently been shown to prevent apoptosis in EC by transferring mitochondria during hypoxic/ischemic stress [89]. Recent data from a model of cigarette smoke-induced lung injury suggests that donor source and age may affect repair via mitochondrial transfer by MSCs [90]. MSCs and EC can exchange mitochondria through tunneling nanotube (TNT)-like structures at the basal level in a bidirectional manner. The mitochondrial exchange occurs with the oxygen–glucose deprivation/reoxygenation stress-induced mitochondrial transfer from MSCs to injured EC, resulting in the rescue of aerobic respiration and the protection of EC from apoptosis [89]. This observation demonstrates that injured HUVECs and co-cultured MSCs create membrane protrusions and extend between each other, creating de novo TNT-like structures, rather than by a mechanism that involves the close contact of adjacent cells and subsequent egress [89].

Stem cell transplantation is expected to change the outcome of a damaged vascular system and the prognosis of patients in the early phase of acute ischemic vascular disease. Investigation of the protective effects of stem cell engraftment via TNT-mediated mitochondrial transfer could provide new insights into the therapeutics of ischemic vascular disease [89].

**Fig. 5:**
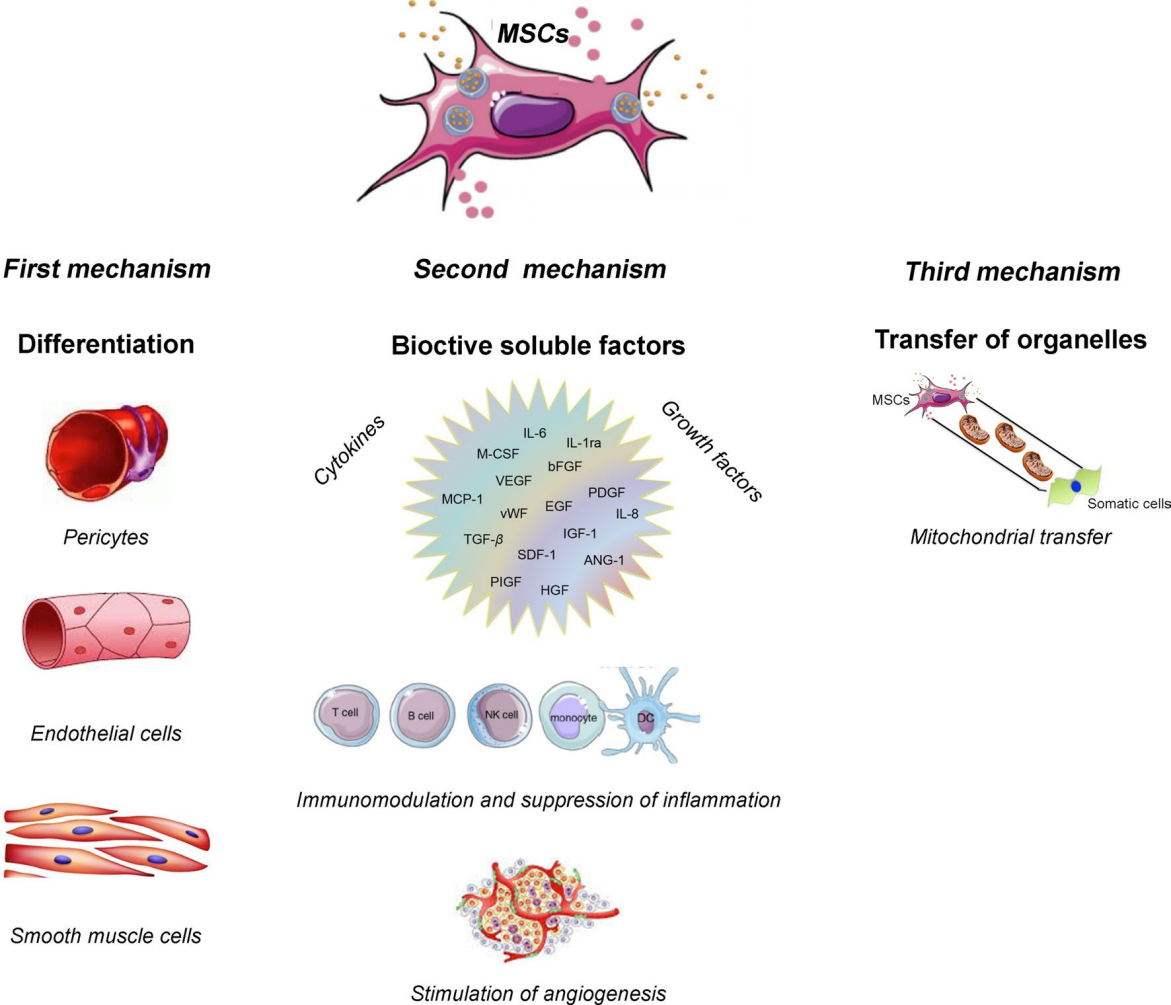
The molecular mechanism associated with the clinical potential of MSCs in CLI

Finally, the molecular mechanisms associated with the angiogenic potential of MSCs are through direct cell differentiation and/or transdifferentiation, cell contact interaction, paracrine signals (immunomodulation effect), and transfer of mitochondria (Fig. 5).

## MSC-based therapy for CLI

A variety of clinical trials in CLI with MSC-based therapy have recently revealed their security profile and therapeutic potential (Table 1). These include, for example, the work by Gupta et al*.*, who conducted a randomized controlled trial in 20 patients with established CLI, presenting Rutherford classification in Categories II-4, III-5, or III-6 with infra-inguinal arterial occlusive disease, and were not suitable or who had undergone failed revascularization treatment. Participants were randomized to receive 200 × 10^6^ allogeneic BM-MSCs or placebo solution (each group *n* = 10), which were injected intramuscularly into the gastrocnemius muscle (40–60 sites, distributed in an area of 10 cm × 6 cm, 1–1.5 cm in depth), and had a 6–24-month follow-up period. The study showed significant improvement in the rest ABPI and ankle pressure in participants treated with cell therapy relative to the patients treated with placebo. Wound healing, pain, and amputation rates were similar in both arms, and no related adverse events related to treatment were reported [91]. In agreement with these outcomes, Lu et al. reported in a comparative study between BM-MSCs (9.3 × 10^8^ ± 1.1 cells) and BM-MNC that BM-MSCs (9.6 × 10^8^ ± 1.1 cells) were injected intramuscularly into the lower limb (20 sites, 3 cm × 3 cm in intervals, 1–1.5 cm in-depth, and 0.5–1 mL BM-MSCs or BM-MNC per site) that BM-MSCs were more potent than BM-MNC. Although BM-MSCs and BM-MNC implantation effectively increased blood flow in all 37 limbs, as assessed by the substantial improvement in rest pain, pain-free walking time, ABPI, TcO_2_, or the formation of new collateral vessels, BM-MSC transplantation was significantly more effective than BM-MNC for the treatment of type 2 diabetic patients with CLI and foot ulcers. There were no acute or chronic serious adverse events related to the BM-MSCs or BM-MNC injection during the 24-week follow-up period. The possible mechanism of therapeutic angiogenesis between the BM-MSCs and BM-MNC in this study was the delivery of angiogenic factors, which promote blood vessel growth and maturation and were detected from both cells in vitro. BM-MSCs from diabetic patients were also found to secrete more VEGF, FGF-2, and angiopoietin-1 than BM-MNC under normoxic and hypoxic conditions [92].

**Table 1:** Evidence of clinical use of stem cells in CLI

Author (year)	Design study and sample size (*n*)	Type of transplant and stem cell source	CLI model	Delivery method	Follow-up time (months)	Therapeutic effect and /or action mechanism
Gupta et al. (2021)[78]	Phase IV, open-label, and multicenter clinical trial (*n* = 50)	Allogeneic BM-MSCs	CLI due to Buerger's disease	Intramuscular and around the ulcer	12	Improvement in rest pain, ankle systolic pressure, and ankle–brachial pressure index with accelerated ulcer healing Anti-inflammatory, immunomodulatory, and angiogenic properties
Norgren et al (2019)[83]	Phase III, randomized, double-blind, multicenter, multinational placebo-controlled, and parallel group clinical trial (*n* = 246)	Allogeneic placental-derived MSCs	CLI Rutherford 5, ineligibility for revascularization or failed revascularization	Intramuscular	12—36	Improvement of amputation-free survival and trends in reduction of pain scores and increase of tissue perfusion Pro-angiogenic, anti-inflammatory, immunomodulating and regenerative properties
Wang et al 2018[9]	Phase I/II, single-center, and open-label clinical trial (*n* = 32)	Allogeneic BM-MSCs and autologous concentrated bone marrow aspirate	CLI with required amputation within next 30 days	Intramuscular	6	Changes in peripheral cytokine signaling, microRNA expression, and pro-angiogenic and inflammatory mononuclear phenotypes Angiogenesis, to decrease muscle fiber apoptosis, and to stimulate re-epithelialization of wound beds
Wijnand et al 2018 [7]	Phase I/II, randomized, double-blind, placebo and controlled clinical trial (*n* = 66)	Allogeneic BM-MSCs	Patients with CLI who are not eligible for conventional revascularization	Intramuscular	6	Improvement mortality, limb status, clinical evolution and changes in pain score
Gupta et al 2017[84]	Phase II, prospective, nonrandomized, open-label, multicenter, and dose-ranging clinical trial	Allogeneic BM-MSCs	CLI due to Buerger’s who had not responded to, or were not eligible for, revascularization	Preclinical: intramuscular (adductor) Clinical: intramuscular (gastrocnemius) and locally [22]	24	Reduction in rest pain, healing of ulcers, improvement in ankle–brachial pressure index and total walking distance No significant difference was observed in the number of collateral vessels and amputation-free survival. Angiogenesis
Tournois et al 2017[85]	No randomization (*n* = 40)	Autologous BM aspirate or peripheral blood	Patients with CLI not suitable for revascularization	Intramuscular	6	Paracrine effect
Bura et al 2014[80]	Phase I consecutively enrolled clinical trial (*n* = 7)	Autologous adipose-derived stroma cell	Diabetic or non-diabetic not suitable candidates for surgery	Intramuscular	6	Increase in the transcutaneous oxygen pressure Improvement ulcers evolution and wound healing Decreased rest pain and number of lesions Differentiation toward endothelial-like cells Paracrine activities
Gupta et al 2013 [78]	Phase I/II, randomized, double-blind, placebo-controlled, multicenter clinical trial (*n* = 20)	Allogeneic BM-MSCs	Controlled diabetic or non-diabetic, failed revascularization or not suitable candidates for surgery	Intramuscular	6 (24)	Increase in the transcutaneous oxygen pressure Improvement in rest pain and ankle–brachial pressure index and ulcer healing
Li et al 2013[86]	Phase II, single-blinded, placebo-controlled clinical trial (*n* = 58)	Autologous bone marrow mononuclear cells	Patients with chronic critical limb ischemia unresponsive to standard revascularization treatment	Intramuscular	6	Improvement in rest pain, ankle–brachial pressure index and ulcer healing No significant differences in the incidence of adverse events among the groups No significant differences in major amputation rates Differentiation into vascular endothelial cells and smooth secretion of vascular growth factors and cytokines Vascular remodeling Neovascularization and collateral vascularization
Das et al 2013 [87]	Phase I, single-center open-label prospective clinical trial (*n* = 10)	Allogeneic BM-MSCs	CLI Rutherford III or more (4 or more)	Intra-arterial	6	Improvement in rest pain and ulcer healing Vasculogenesis that occurs mainly in smaller vessels
Mohammadzadeh et al. 2013 [88]	Randomized, controlled, and parallel clinical trial (*n* = 21)	Autologous peripheral blood MSCs mobilized by G-CSF	Diabetic, angioplasty failure (or else could not benefit from angioplasty)	Intramuscular	3	Improvement in amputation rate, pain-free walking distance and wound healing. Differentiation and incorporation into the endothelial cells lining the blood vessels and neovascularization blood flow
Powell et al 2012 [82]	Phase II, double-blind, placebo-controlled, randomized clinical trial (*n* = 72)	Ixmyelocel T: (Autologous MNC, MSC, activated macrophages)	Diabetic and non-diabetic, not revascularizable	Intramuscular	12	Significant reduction in the risk of treatment failure in the Ixmyelocel T-treatment group The occurrence of adverse events and serious adverse events was similar between the two treatment groups No reported amputation-free survival
Lu et al. 2011[79]	Phase I /II double-blind, randomized, placebo-controlled clinical trial (*n* = 41)	Autologous BM-MSCs or bone marrow mononuclear cells	Type 2 diabetic patients with bilateral critical limb ischemia	Intramuscular	6	Improvement in ulcer healing rate, painless walking time and ankle–brachial pressure index. No significant difference in amputation Increase in the transcutaneous oxygen pressure. Significantly increased collateral vessels (increased scores > 2) greater in MSCs group. Release of angiogenic factors Increased blood flow
Lasala et al 2010 [81]	Phase I, single-center, nonrandomized, single-group assignment clinical trial (*n* = 10)	Autologous BM-derived mononuclear and BM-MSCs	Severe limb ischemia (Fontaine stages 2B to 4), non-revascularizable	Intramuscular	6 (10)	Improvement, painless walking time, ankle–brachial pressure index and physical functioning Significant formation of new blood vessels. Paracrine effect therapeutic. Vasculogenesis. Enhancement of blood flow Collateral vessel formation
Kim et al. 2006 [89]	Clinical trial (*n* = 27)	Allogeneic MSCs derived from umbilical cord blood or mobilize endothelial progenitor cells (EPCs) from bone marrow	CLI Buerger’s disease	Intramuscular and subcutaneous (adjacent lesions)	4	Increased capillary formation on the affected lesions and decreased vascular resistance and arteriogenesis Paracrine factors(cytokines and growth factors) No side effects

MSCs derived from other sources have also shown their angiogenic potential in CLI. In the study by Bura et al*.*, autologous AD-MSCs (1 × 10^8^ cells) were intramuscularly administrated (15 sites for each muscle with the use of a standard grid) in seven diabetic and non-diabetic patients who were not suitable for vascular or endovascular surgery. No adverse event was associated with autologous AD-MSC transplantation during the follow-up. Six months after cell transplantation, a significant increase in TcPO_2_, reduction in rest pain, and wound healing were also observed. Nevertheless, no ABPI improvement or change in CLI grade was achieved [93]. Similar results have been reported with MSCs derived from other tissues, such as the placenta or umbilical cord.

Other studies have proved the security and efficacy profile of combined cellular products; for example, Lasala et al. evaluated the intramuscular administration of a combination of autologous bone marrow-derived EPCs and BM-MSCs. No adverse events were reported during the clinical trial. At six-month follow-up, there was an increase in ABPI, walking time, pain relief, and physical functioning. Although changes in TcPO_2_ were not statistically significant, the formation of new blood vessels was confirmed by angiography, suggesting that these may correspond to collateral small vessels which may improve perfusion outcomes but do not affect all clinical values [94].

On the other hand, Powell et al. evaluated Ixmyelocel T-treatment, which is a patient-specific, expanded, multicellular therapy containing autologous BM-MNC, BM-MSCs, and activated macrophages. This study was a Phase 2, double-blind, placebo-controlled, randomized trial conducted to assess both the safety and efficacy of intramuscular injections of Ixmyelocel T-treatment (*n* = 48) versus placebo (*n* = 28) in patients with CLI and no options for revascularization. This trial provides encouraging evidence that treatment with Ixmyelocel T is safe and beneficial in treating lower extremity CLI in a “no-option” population. Efficacy outcomes showed a statistically significant improvement in time to treatment failure (TTF) and in amputation-free survival (AFS) in Ixmyelocel T-treated patients relative to controls. The treatment effect for both TTF and AFS was even more pronounced in patients who entered the trial with baseline wounds, suggesting greater efficacy in more severe and advanced diseases. These results suggest that treatment with Ixmyelocel T is a promising treatment option for patients with CLI who are unable to undergo revascularization [95].

## Conclusions

Overall, this review has demonstrated the fascinating angiogenic and regenerative properties of MSCs, which provide a functional advantage over other conventional strategies. Research in this area has been limited by the recent improvement in surgical techniques and the rapid progression of ischemia, however, leading to amputation in some patients, which hinders the recruitment of suitable candidates.

Our search of *clinicaltrials.com* yielded 26 clinical trials involving the use of MSCs in the treatment of CLI, of which 15 are currently ongoing. Although available clinical studies demonstrate that vascular remodeling and blood flow restoration encourages MSC-based therapy in the treatment of CLI patients, more multicenter clinical trials are required. Further research is also needed to strengthen the evidence in favor of these promising findings and elucidate aspects such as the best route of administration, the best MSC sources, optimal culture conditions, the local environment affecting their performance and action, and the special markers modulating the angiogenic response to propose the more optimized therapeutic strategies.

MSC-based therapy is on the way to becoming a feasible therapeutic option in the context of failed revascularization or non-revascularizable disease. MSC transplantation for CLI relies on the ability of MSCs to maintain vascularization and angiogenesis. Injected cells can act beneficially by improving local angiogenesis (either through the maturation of endothelial progenitors or through the secretion of angiogenic mediators), or by transducing cytoprotective signals that preserve tissue structure.

## Data Availability

The authors confirm that the data supporting the findings of this study are available within the article [and/or] its supplementary materials.

## Abbreviations

AD: Adipose tissue
ANG-1: Angiopoietin-1
AFS: Amputation-free survival
ABPI: Ankle–brachial pressure index
αSMA: Anti alpha smooth muscle actin
a-BM-SC: Autologous bone marrow stem cells
BM: Bone marrow
BM-MSCs: Bone marrow-derived MSCs
BrdU: Bromodeoxyuridine
CFSE: Carboxyfluorescein diacetate succinimidyl ester
CXCL: C-X-C motif chemokine ligand
CFU-F: Colony-forming unit-fibroblasts
CM: Conditioned medium
CLI: Critical limb ischemia
DBCO: Dibenzyl cyclooctyne
EC: Endothelial cell
EPCs: Endothelial progenitor cells
EGF: Epidermal growth factor
FBS: Fetal bovine serum
FGF: Fibroblast growth factor
HGF: Hepatocyte growth factor
hAD-MSCs: Human adipose-derived MSCs
HLA: Human leukocyte antigen
hPMSCs: Human placenta-derived mesenchymal stem cells
HIF-1: Hypoxia-inducible factor-1
IGF-1: Insulin growth factor-1
IV: Intravenous
IA: Intra-arterial
IL: Interleukin
IL-1ra: Interleukin-1 receptor antagonist
ILB4^+^: Isolectin B4
IGFBP: Insulin-like growth factor binding protein
LDPI: Laser Doppler perfusion image
Luc^+^mBM-MSCs: Luciferase-labeled mBM-MSCs
M-CSF: Macrophage colony-stimulating factor
MMP: Matrix metalloproteinase
MSCs: Mesenchymal stem cells
MCP-1: Monocyte chemoattractant protein-1
NOD-SCID: Non-obese diabetic/severe combined immunodeficiency
PAD: Peripheral arterial disease
PDGF: Platelet-derived growth factor
rBM-MSCs: Rat bone marrow-derived MSCs
rBM-MNC: Rat bone marrow-derived mononuclear cells
RT-PCR: Reverse transcription polymerase chain reaction
SDF-1: Stromal-derived factor-1
TGF-*β*: Transforming growth factor-beta
TTF: Time to treatment failure
TIMP: Tissue inhibitor of metalloproteinases
TcPO_2_: Transcutaneous partial pressure of oxygen
TNT: Tunneling nanotube
UCB: Umbilical cord blood
UA-hAD-MSCs: Undifferentiated adipose MSCs
US: United States
VCAM-1: Vascular cell adhesion molecule-1
VEGF: Vascular endothelial growth factor
VLA-4: Very late antigen-4
VHL: Von Hippel–Lindau
WJ: Wharton’s jelly
vWF: Willebrand factor
